# Characteristics of Harmonic Indexes of the Arterial Blood Pressure Waveform in Type 2 Diabetes Mellitus

**DOI:** 10.3389/fbioe.2020.00638

**Published:** 2020-07-08

**Authors:** Chen-Kai Liao, Jaw-Shiun Tsai, Liang-Yu Lin, Si-Chen Lee, Chun-Fu Lai, Te-Wei Ho, Feipei Lai

**Affiliations:** ^1^Graduate Institute of Biomedical Electronics and Bioinformatics, National Taiwan University, Taipei, Taiwan; ^2^Lao De Yan Traditional Chinese Medicine Clinic, New Taipei City, Taiwan; ^3^Department of Family Medicine, National Taiwan University Hospital, Taipei, Taiwan; ^4^Division of Endocrinology and Metabolism, Department of Medicine, Taipei Veterans General Hospital, Taipei, Taiwan; ^5^Faculty of Medicine, National Yang-Ming University, Taipei, Taiwan; ^6^Department of Electrical Engineering, National Taiwan University, Taipei, Taiwan; ^7^Department of Internal Medicine, National Taiwan University Hospital, Taipei, Taiwan; ^8^Department of Surgery, National Taiwan University Hospital, Taipei, Taiwan; ^9^College of Medicine, National Taiwan University, Taipei, Taiwan; ^10^Department of Computer Science and Information Engineering, National Taiwan University, Taipei, Taiwan

**Keywords:** diabetes mellitus, blood pressure, hemodynamic, stiffness, harmonic, fast Fourier transform

## Abstract

Type 2 diabetes mellitus (T2DM) is an important public health issue worldwide. T2DM correlates with cardiovascular disease. Arterial stiffness is also a key factor that can be thought of as a surrogate marker. Nevertheless, it was unclear which harmonic indexes of blood pressure waveforms (BPWs) from subjects' radial artery pulses would be affected by T2DM. Therefore, the objective of this study was to investigate whether and how harmonic indexes can be used to discriminate hemodynamic differences between patients with T2DM and non-T2DM. This helps us to build objective results no matter who conducts the examination instead of pulse diagnosis in traditional way. We enrolled T2DM and non-T2DM patients as experimental and control groups, respectively, from the Department of Family Medicine in the National Taiwan University Hospital and the Department of Internal Medicine in Taipei's Veterans General Hospital from December 2017 to January 2019. ANSWatch® Model TS-0411 was used to capture the BPWs. Amplitude proportions (C_n_ values) were calculated from harmonics 1–10 of the BPW using fast Fourier transform. Thirty-two T2DM and 15 non-T2DM patients were enrolled. T2DM patients had significant differences in C_1_ (*p* = 0.031) and C_5_ (*p* = 0.041). The study suggests that analyzing the harmonic characteristics of non-invasively measured BPW of radial artery may be a potential and easy-to-perform approach to discriminate T2DM-induced hemodynamic changes.

## Introduction

Diabetes mellitus (DM) is one of the most important public health issues worldwide. The vast majority of diabetes patients have type 2 diabetes mellitus (T2DM). In 2014, it was estimated that 422 million adults above 18 years of age suffered from DM worldwide (WHO, 2016). The global prevalence of DM in 1980 was 4.7%, but it had risen to 8.5% by 2014. Being overweight or obese is strongly linked to diabetes occurrence (WHO, 2016). The trends in the complications of diabetes are cardiovascular disease, chronic kidney disease, loss of vision and lower extremity amputations (Wolf and Ritz, 2003; Radbill et al., 2008; Tripathi and Yadav, 2013; WHO, 2016). DM is a serious threat to a person's health and quality of life.

T2DM patients have higher aortic stiffness (Taniwaki et al., 1999; Kimoto et al., 2003; De Angelis et al., 2004) and higher risk for cardiovascular morbidity and mortality. Large artery stiffness is increased in patients with DM (Cameron et al., 2003; Kimoto et al., 2006; Prenner and Chirinos, 2015). Macrovascular complications in patients with DM involve coronary arteries (Haffner et al., 1998), cerebrovascular vessels (Sarwar et al., 2010), and peripheral arteries (Lange et al., 2004). Kimoto et al. (2006) demonstrated that DM was correlated to pulse wave velocity of the central arteries-heart-carotid and heart-femoral segments significantly. Palombo and Kozakova (2016) showed that stiffness can be considered a surrogate marker of cardiovascular risk. Sarwar et al. (2010) also showed that DM confers about two times the excess risk for vascular diseases, independently from other conventional risk factors.

The pulse examination of traditional Chinese medicine is a unique diagnostic technique absent in Western medicine. It has been implemented for thousands of years throughout the eastern world. Nowadays, some studies in how to digitalize pulse objectively had been proposed (Wang et al., 2010). Physicians can use modern devices instead of traditional pulse examination to collect body internal information from patients' radial artery pulses. This helps us to build objective results no matter who conducts the examination.

Because the transmission of arterial pressure pulse and therefore pulse contour can be affected by changes in vascular conditions along vessels and in vascular beds, blood pressure waveform (BPW) indexes may provide information regarding changes in vascular properties and blood flow (Hsu et al., 2014). Time-domain BPW indexes, including the augmentation index and pulse wave velocity, have been used to detect vascular changes in various cardiovascular diseases (Safar et al., 2003; Avolio et al., 2010; Tuttolomondo et al., 2011). BPW frequency-domain analysis is another way to describe pulse contour (Milnor, 1989). Variation in spectral parameters of BPW has also been proposed to help monitor these properties of blood vessels in basic studies (Taylor, 1966; Li et al., 1981; Wang, 1986; Milnor, 1989; Ming-Yie et al., 2003; Wang et al., 2010) and diseases (e.g., breast cancer, polycystic ovary syndrome, and stroke) in humans (Hsiu et al., 2013; Hsu et al., 2014; Chen et al., 2017). Among the various frequency-domain analysis methods, harmonic analysis is particularly appropriate for measuring BPW signals (Wei and Chow, 1985), because heartbeats are quasiperiodic. Nevertheless, it is unknown as to whether and how harmonic indexes can be used to discriminate between patients with T2DM and those without T2DM. Therefore, the objective of this study was to investigate whether and how harmonic indexes can be used to discriminate hemodynamic differences between patients with T2DM and non-T2DM.

## Methods

### Subjects

Forty-seven subjects over 40 years old were recruited from the Department of Family Medicine in National Taiwan University Hospital and the Department of Internal Medicine in Taipei Veterans General Hospital from December 2017 to January 2019. Patients with and without T2DM were enrolled. Those with coronary artery disease, hypertensive heart disease, heart failure, valvular heart disease, carditis, any type of cancer, and cerebrovascular accident were excluded, because they might have influenced the aspect of arterial waveform and led to extreme deviations.

The T2DM group included 32 patients with T2DM who were diagnosed by their attending physicians and had a HbA1c ≧ 6.5%. Fifteen subjects who were not diagnosed with T2DM and had a HbA1c <6.0% were classified as the non-T2DM group. After receiving approval from the Research Ethics Committee, National Taiwan University Hospital (REC permit No. 1063704895) and the Institutional Review Board, Taipei Veterans General Hospital (IRB permit No. 1064903562), all subjects signed consent forms before the study. All subjects were voluntarily recruited, were Taiwanese, and most lived in the greater Taipei area.

### Study Procedures

**Figure 1:**
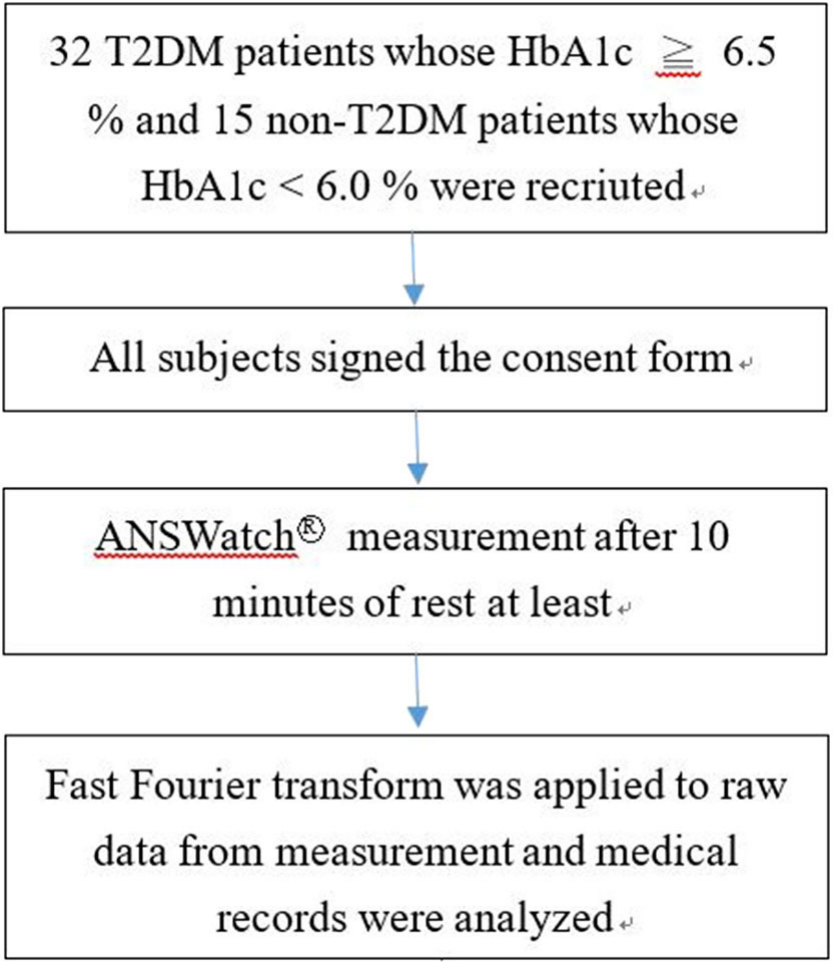
Flow chart of the study.

We ensured that the subjects from the outpatient department met our selection criteria before enrolment. The subjects were recruited during outpatient department time that was 9 a.m.−12:30 p.m. and 2–6 p.m. The measurement was conducted in a room maintained at 23–25°C. We used the ANSWatch® Model TS-0411 (Taiwan Scientific Corporation, medical device product registration number 001525, permitted by the Department of Health, Taiwan) to collect pulse information from patients after they took a break for at least 10 min and signed the consent form. All participants were measured at sitting position and were asked to relax during the measurement to avoid big motions. The flow chart of this study is shown in Figure 1.

### Apparatus and Harmonic Analysis

**Figure 2:**
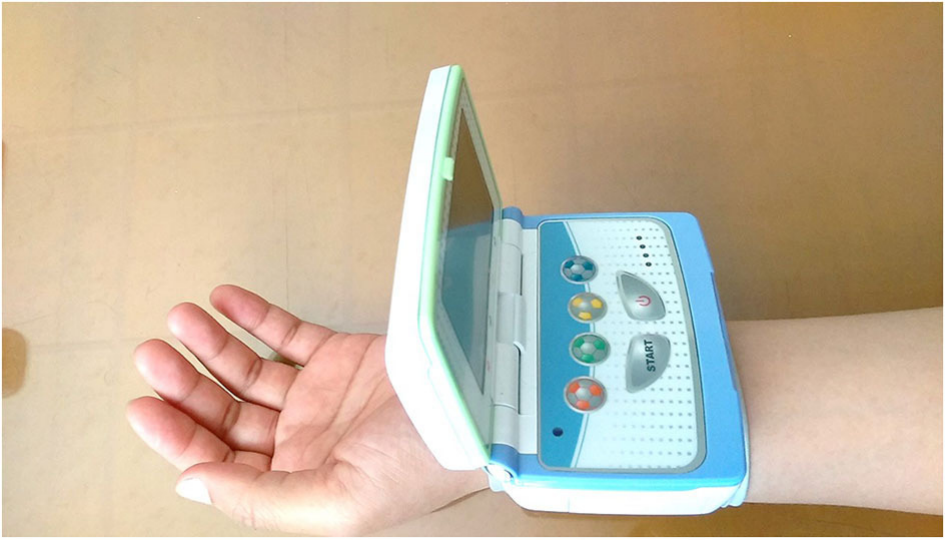
ANSWatch®.

Radial artery pulse pressure were recorded on the left wrist using a pulse wave analyzer ANSWatch® Model TS-0411 that has demonstrated accuracy and repeatability (Sun et al., 2011) (Figure 2). ANSWatch® includes a contact-type piezoelectric sensor that measures the pressure index of the radial artery pulse at a 500 Hz sampling frequency for 5 min and obtains 150,000 raw data points per test. Raw data measured by ANSWatch® can be used to calculate several parameters, including systolic blood pressure, diastolic blood pressure, heart rate, low frequency, high frequency, irregular heartbeats, and heart rate variability (Liang et al., 2009; Wu et al., 2009; Chang and Shen, 2011; Lin, 2013; Wang et al., 2013; Lee et al., 2016). Measurement data were downloaded to a notebook and ANSWatch® Manager Pro software (Taiwan Scientific Corporation, Taipei, Taiwan) was used to obtain raw data.

**Figure 3:**
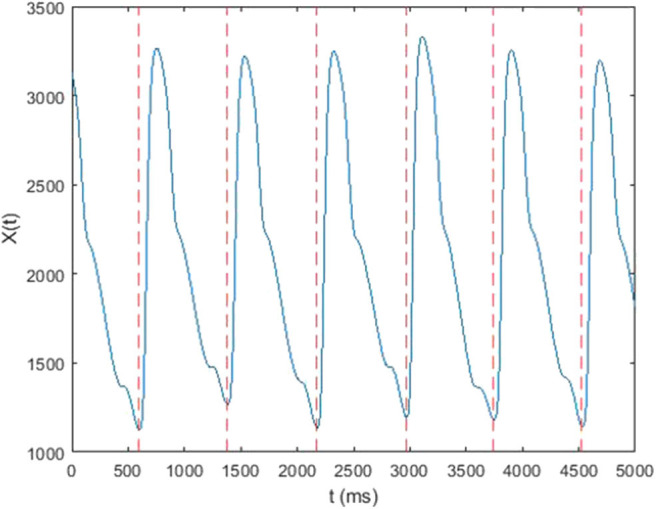
Raw data cut vs. time.

**Figure 4:**
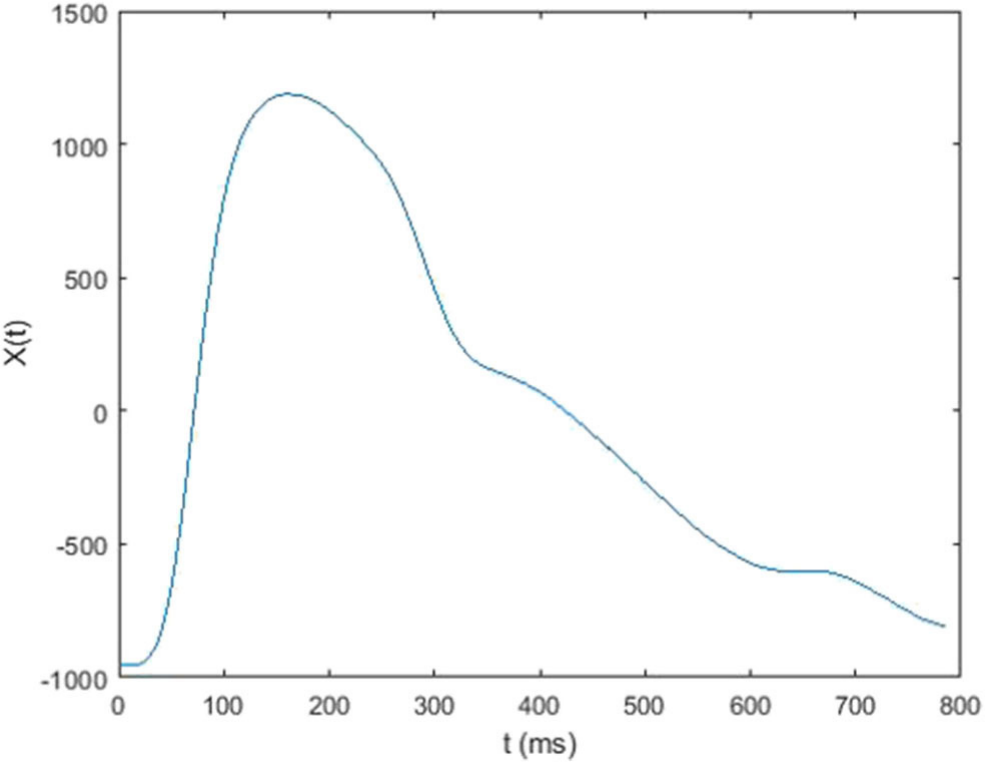
A single wave data (alternating signal) vs. time.

**Figure 5:**
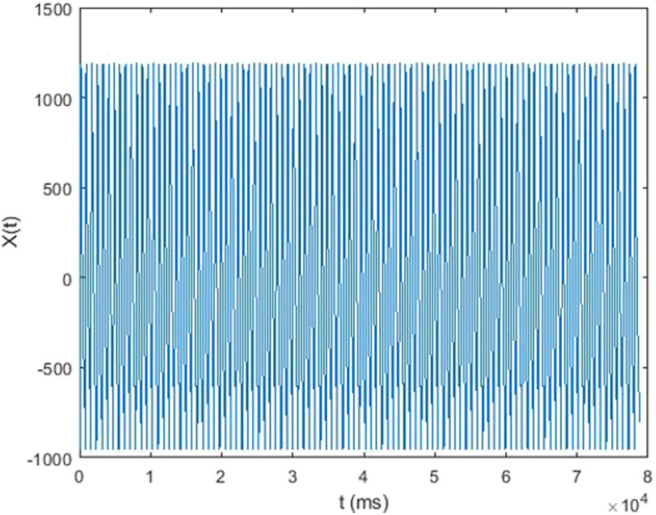
Repeated single wave (alternating signal) vs. time.

**Figure 6:**
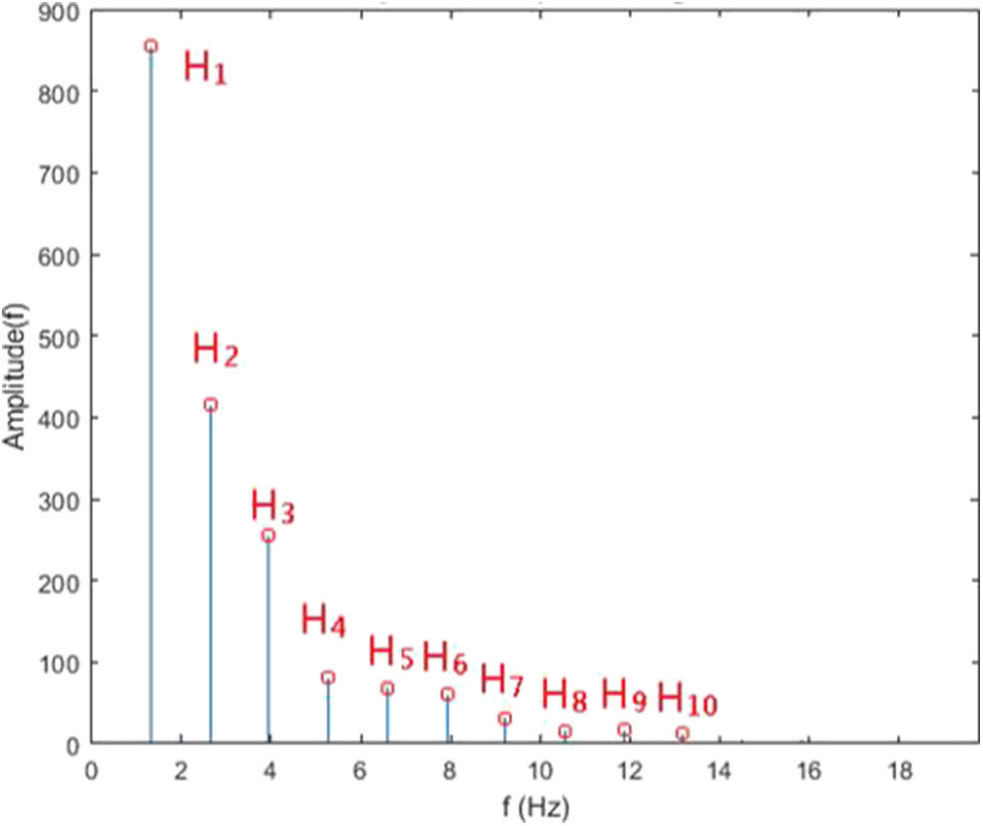
FFT amplitude of repeated single wave.

In this study, each 5-min raw data was divided into subset of around 300–500 single waves (Figure 3). In each subset, mean value of each single wave data was subtracted from corresponding single wave data to retrieve the alternating signal of pressure pulse (Figure 4). After that, every single wave was repeated one hundred times independently to generate periodic waves applicable to Fourier transform (Figure 5). Fast Fourier transform function of the software MATLAB (MathWorks, Natick, MA, US) was then applied to each periodic waves data to retrieve values of ten harmonic coefficients, H_n_, of the Fourier series. The first harmonic coefficients, H_1_, are defined as the maximum coefficient in the frequency domains, while the other nth harmonic coefficients were at the frequencies of *n* times the frequency of corresponding first harmonic coefficient (Figure 6). Coefficient-wise average values of these harmonic coefficients of repeated waves from raw data of the same measurement were calculated, and named A_n_, where *n* = 1–10. These A_n_ values were then transformed into amplitude proportions (C_n_). C_n_ was defined by the following equation:

$$\begin{array}{l} C_{\text{n}}=A_{\text{n}}/A_{0}, \end{array}$$

*n* = 1–10, which contained more than 95% of the energy of the alternating signal of pressure pulse (Milnor, 1989), A_0_ is the average value of pulse wave raw data in time domain and A_n_ is the average of n^th^ harmonic coefficient of the Fourier series of the repeated single wave from original pulse wave data.

### Statistical Analysis

We used SPSS 18 (SPSS Inc., Chicago, IL, US) to carry out all statistical analyses. Variables were expressed as means with associated standard deviations. Statistical significance was set at *p* < 0.05 using two-tailed Student's *t*-test or chi-square test.

## Results

Table 1 shows patients' clinical and demographic characteristics. There were no differences in terms of sex, age, diastolic blood pressure, systolic blood pressure, heart rate or smoke habits between the experimental and control groups. However, body mass index was higher in T2DM patients (*p* = 0.032). Any increase in body mass index above normal weight levels is associated with an increased risk of being diagnosed with DM-related complications (Gray et al., 2015); this is aligned with our findings in this study.

**Table 1:** Clinical and demographic characteristics of T2DM and non-T2DM.

**Variables**	**T2DM**	**Non-T2DM**	***P*-value**
*N*	32	15	
Age (years)	67.41 ± 10.59	66.47 ± 13.13	0.794
Sex			0.401*
Male	17 (53.13%)	6 (40.00%)	
Female	15 (46.88%)	9 (60.00%)	
HbA1c (%)	7.58 ± 1.06	5.57 ± 0.22	<10^−8^
Body mass index (kg/m^2^)	25.95 ± 5.00	22.90 ± 2.59	0.032
Systolic blood pressure (mm Hg)	123.25 ± 12.97	119.73 ± 13.80	0.400
Diastolic blood pressure (mm Hg)	78.13 ± 8.48	77.93 ± 7.57	0.941
Heart rate (bpm)	77.50 ± 12.22	74.80 ± 7.24	0.434
Smoke habit			0.756*
Current	2 (6.25%)	0 (0%)	
Past	1 (3.13%)	1 (6.67%)	

*Data are expressed as mean ± standard deviation values. All p-values were calculated by two-tailed Student's t test except asterisk symbol by chi-square test*.

**Figure 7:**
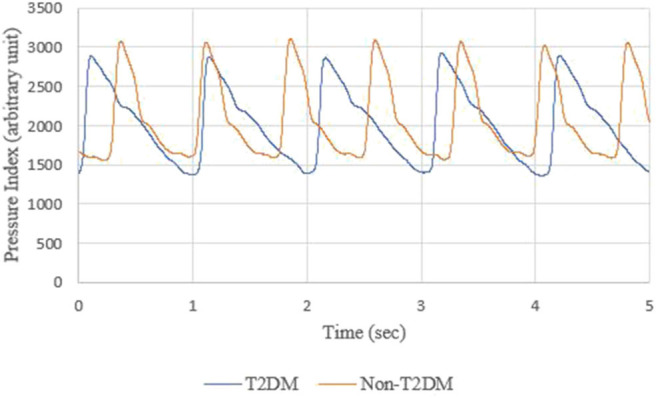
BPWs of non-T2DM and T2DM patients in time domain.

The BPWs of non-T2DM and T2DM patients in the time domain are shown in Figure 7.

**Figure 8:**
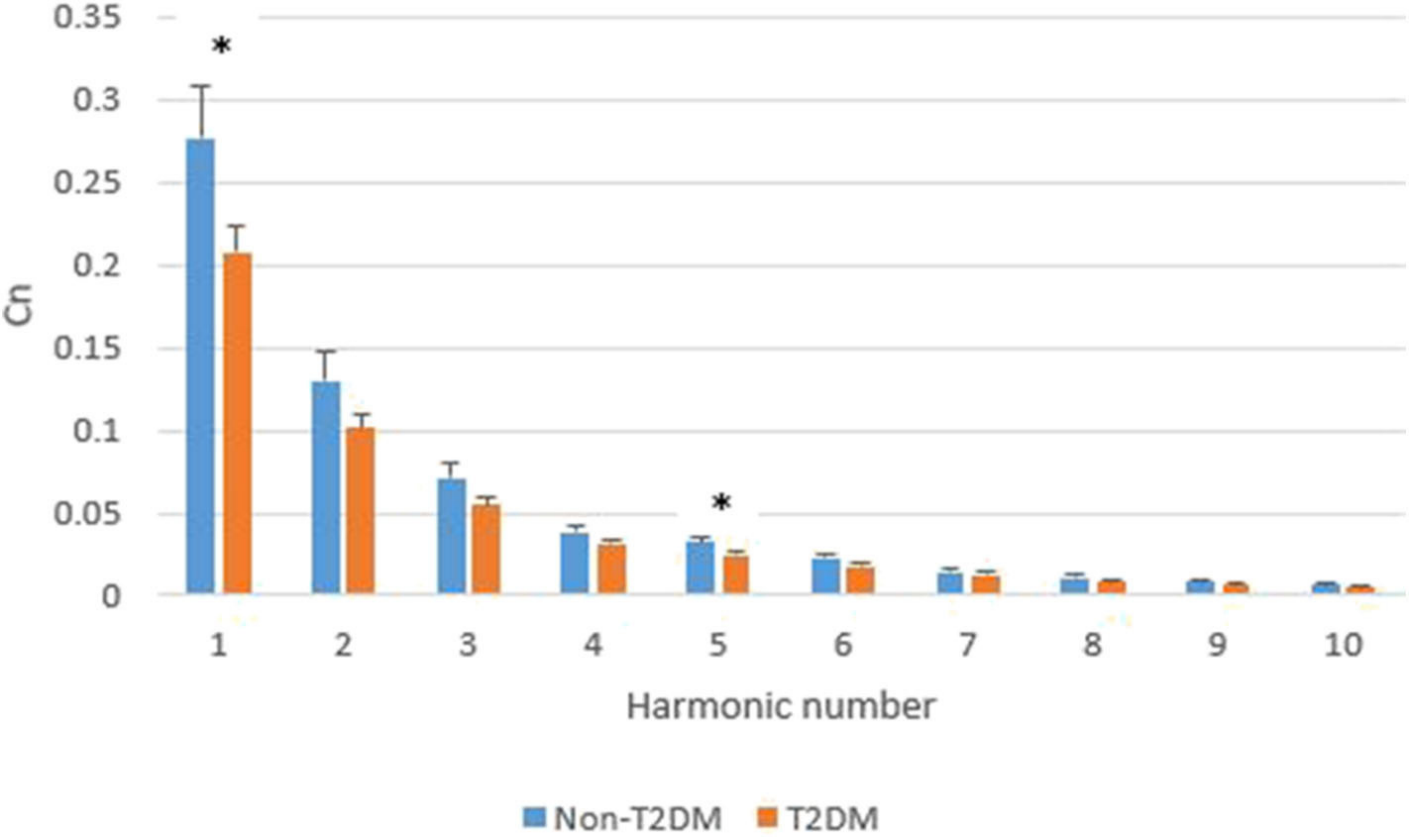
Comparison of C_n_ in T2DM and non-T2DM patients. The data are represented as mean and standard error values for each harmonic. The asterisk indicates the means of C_n_ in the two groups differ significantly (*p* < 0.05) using the two-tailed Student's *t*-test.

Figure 8 shows the comparison of harmonic amplitude proportions of radial pressure waves in T2DM and non-T2DM patients. All C_n_ in the T2DM group were lower than non-T2DM group. Among C_n_, the C_1_ and C_5_ in the T2DM group were significantly lower than non-T2DM group (C_1_, *p* = 0.031, C_5_, *p* = 0.041).

## Discussion

In our study, T2DM patients had different harmonic characteristics from non-T2DM patients. Within the scope of our knowledge, this is the first research study showing the characteristics of harmonic indexes of arterial BPWs in T2DM patients.

Table 2 shows the correlation analysis between harmonics of BPW and other physiological parameters to see whether there were any inter-relationships between them. There was no high correlation (>0.7) between each other except a part of C_n_. Since features of biomedical signals usually can be represented in several sub-bands, we found higher correlation values located in the diagonal direction in Table 2.

**Table 2:** The correlation analysis between harmonics of BPW and other physiological parameters (yellow marks represent high correlation which is >0.7).

	**Sex**	**BMI**	**Age**	**HbA1c**	**Smoker**	**SYS**	**DIA**	**HR**	**C1**	**C2**	**C3**	**C4**	**C5**	**C6**	**C7**	**C8**	**C9**	**C10**
Sex	1	0.384	−0.137	0.037	0.309	0.075	0.183	0.084	−0.217	−0.085	0.022	0.015	0.008	0.048	0.084	0.060	0.074	0.121
BMI	0.384	1	−0.184	0.251	−0.039	0.069	0.248	0.207	−0.295	−0.220	−0.070	−0.029	−0.101	−0.005	0.070	0.031	0.076	0.117
Age	−0.137	−0.184	1	0.074	−0.104	0.073	−0.313	−0.424	0.337	0.239	0.317	0.367	0.312	0.351	0.401	0.439	0.463	0.462
HbA1c	0.037	0.251	0.074	1	0.074	0.163	0.062	0.265	−0.234	−0.225	−0.189	−0.132	−0.190	−0.149	−0.060	−0.102	−0.087	−0.044
Smoker	0.309	−0.039	−0.104	0.074	1	−0.103	0.060	−0.040	−0.327	−0.309	−0.230	−0.256	−0.330	−0.241	−0.201	−0.229	−0.213	−0.145
SYS	0.075	0.069	0.073	0.163	−0.103	1	0.582	−0.087	−0.268	−0.239	−0.232	−0.123	−0.099	−0.096	0.014	0.052	0.092	0.133
DIA	0.183	0.248	−0.313	0.062	0.060	0.582	1	0.206	−0.397	−0.326	−0.302	−0.216	−0.198	−0.238	−0.204	−0.176	−0.139	−0.115
HR	0.084	0.207	−0.424	0.265	−0.040	−0.087	0.206	1	−0.175	−0.105	−0.232	−0.253	−0.190	−0.353	−0.406	−0.377	−0.385	−0.404
C1	−0.217	−0.295	0.337	−0.234	−0.327	−0.268	−0.397	−0.175	1	0.942	0.853	0.819	0.855	0.765	0.652	0.625	0.581	0.490
C2	−0.085	−0.220	0.239	−0.225	−0.309	−0.239	−0.326	−0.105	0.942	1	0.923	0.823	0.861	0.776	0.621	0.557	0.516	0.418
C3	0.022	−0.070	0.317	−0.189	−0.230	−0.232	−0.302	−0.232	0.853	0.923	1	0.897	0.898	0.905	0.756	0.666	0.646	0.556
C4	0.015	−0.029	0.367	−0.132	−0.256	−0.123	−0.216	−0.253	0.819	0.823	0.897	1	0.963	0.940	0.910	0.846	0.800	0.736
C5	0.008	−0.101	0.312	−0.190	−0.330	−0.099	−0.198	−0.190	0.855	0.861	0.898	0.963	1	0.936	0.874	0.845	0.801	0.718
C6	0.048	−0.005	0.351	−0.149	−0.241	−0.096	−0.238	−0.353	0.765	0.776	0.905	0.940	0.936	1	0.942	0.876	0.856	0.789
C7	0.084	0.070	0.401	−0.060	−0.201	0.014	−0.204	−0.406	0.652	0.621	0.756	0.910	0.874	0.942	1	0.961	0.933	0.905
C8	0.060	0.031	0.439	−0.102	−0.229	0.052	−0.176	−0.377	0.625	0.557	0.666	0.846	0.845	0.876	0.961	1	0.979	0.951
C9	0.074	0.076	0.463	−0.087	−0.213	0.092	−0.139	−0.385	0.581	0.516	0.646	0.800	0.801	0.856	0.933	0.979	1	0.982
C10	0.121	0.117	0.462	−0.044	−0.145	0.133	−0.115	−0.404	0.490	0.418	0.556	0.736	0.718	0.789	0.905	0.951	0.982	1

**Figure 9:**
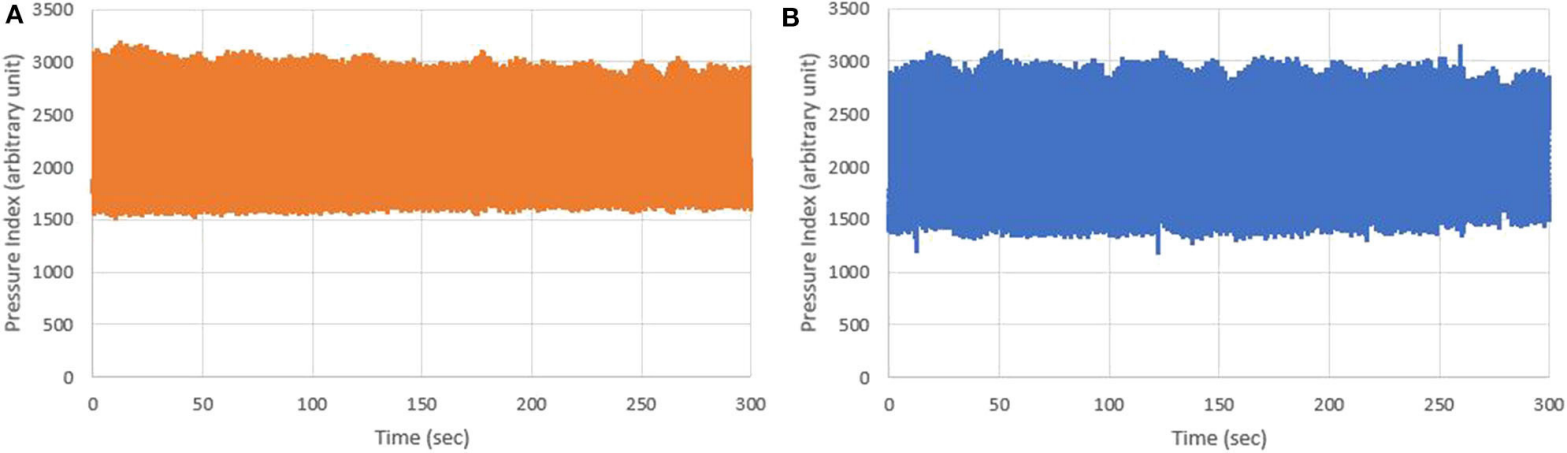
**(A)** Five-minute BPWs of non-T2DM patients; and **(B)** Five-minute BPWs of T2DM patients measured by ANSWatch®.

The BPWs in time domain cannot be digitized to get the difference from Figure 7, but harmonic analysis does not have this problem. Furthermore, although BPW indexes in time domains can help monitor transmission conditions of arterial pressure pulses, these indexes can be interfered by noise, body movement and other factors, resulting in lower accuracy. The shapes of time-domain BPWs were influenced due to parts of these factors in Figures 9A,B.

Harmonic analysis of BPW provides another way to monitor changes in vascular properties including detecting changes in arterial elasticity by monitoring changes in harmonic amplitudes (Latham et al., 1985; Hsiu et al., 2012). The present study showed that the harmonic amplitude proportions of BPWs in T2DM patients differ from those in non-T2DM patients. It implies that harmonic analysis could be used to develop non-invasive indexes to discriminate hemodynamic differences in arterial elasticity induced by T2DM.

**Figure 10:**
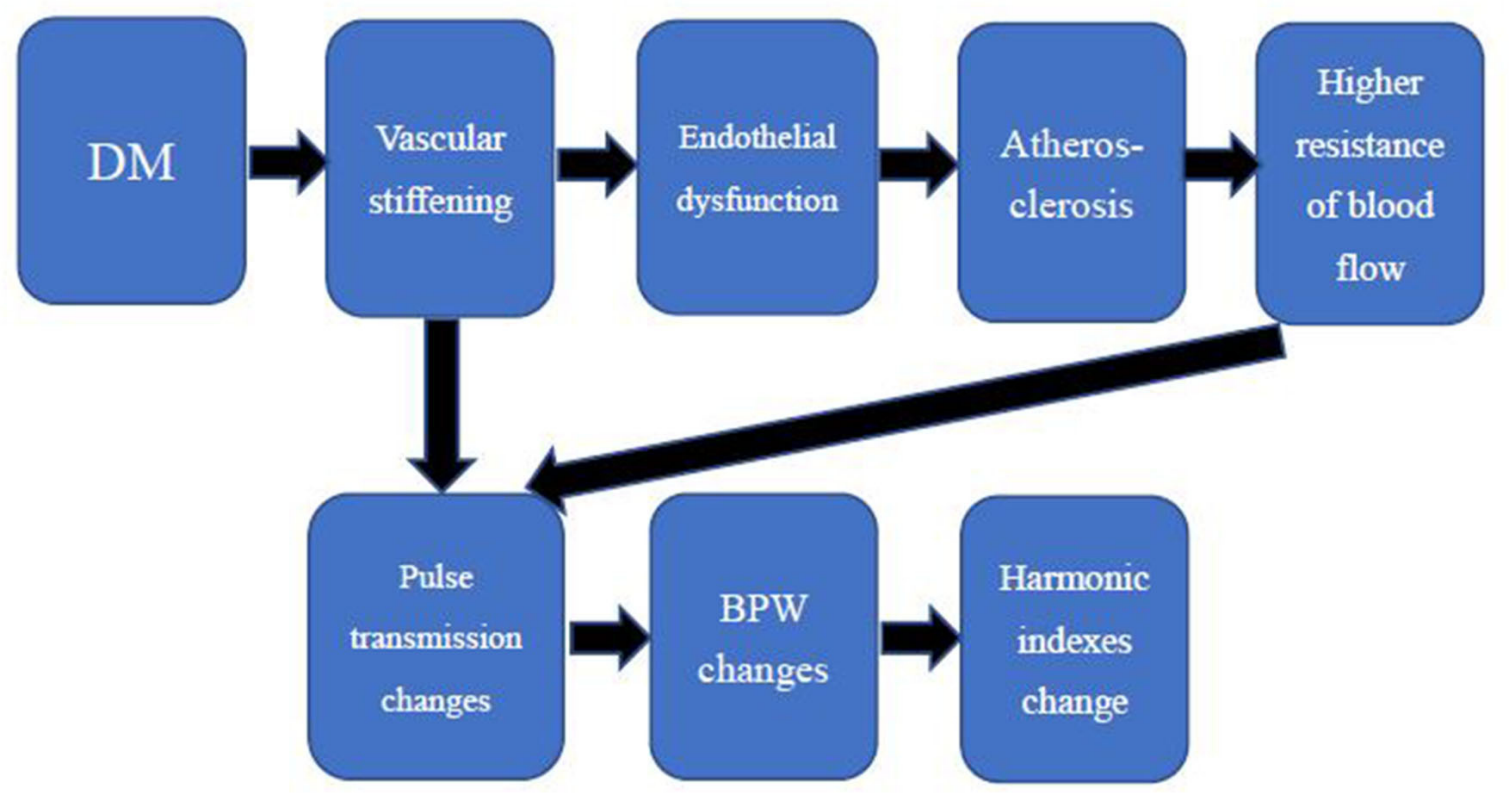
The changing process of harmonic indexes in diabetic patients.

Several studies have shown that DM results in early arterial stiffness because it is an independent risk factor for structural and functional damage to the arterial wall (van der Meer et al., 2007; Naka et al., 2012; Smulyan et al., 2016). Vascular stiffening results in higher arterial pulse pressure and pulsatile shear, exacerbating endothelial dysfunction (Zieman et al., 2005), a key antecedent and modulator of atherosclerosis that has been displayed in prediabetes (Su et al., 2008). DM is one of the major risk factors of atherosclerosis^1^. Plaque of the arteries leads to higher resistance of blood flow because it narrows the cross-sectional area of vessels. Since the elasticity of arterials and resistance of blood flow change, pulse transmission changes and then results in different BPWs and harmonic indexes. The relationships are shown in Figure 10. The high frequency of harmonic components transmitted smoother because artery stiffness was increased in patients with DM. The decay of high frequency of harmonic components was less, then the proportion of low frequency of harmonic components decreased. It was supposed that it was the reason why T2DM group had significant lower value in C_1_ (Figure 8). The elasticity of arterials and resistance of blood flow are correlated with C_1_ and C_5_ in this study. These factors are independent of other cardiovascular diseases since subjects with other cardiovascular disease were excluded as mentioned in the method of this study. Besides, it needs further research to know whether there are other determinant factors of C_1_ and C_5_. A change in the index could be partly attributed to T2DM-induced hemodynamic change. This implies that change in the index could represent change in arterial stiffness and resistance of blood flow, which is in line with T2DM arterial stiffening. The current study might be developed to provide an easier screening tool for measuring these indexes in T2DM patients instead of measuring patients' arterial stiffness in the future.

Different diseases can lead to different harmonic distributions in hemodynamics. In previous studies, stroke patients had significant amplitude changes in the 2nd, 5th, 6th, and 7th harmonics (Chen et al., 2011). Patients with liver cirrhosis had significant changes in the 1st, 3rd, and 6th harmonics (Lu et al., 1999). Patients with polycystic ovary syndrome had significant changes in the 1st and 4th harmonics (Hsu et al., 2014). The 1st and 2nd harmonics were significantly larger in patients with breast cancer (Chen et al., 2017). The 1st and 5th harmonic were significantly smaller in T2DM patients in this study. Those different harmonic characteristics further imply that the harmonic distribution of the BPW might help distinguish patients with different diseases.

Young et al. (1989, 1992) showed that the second harmonic component of BPW was prominently lower while ligating the renal artery and the third harmonic component of BPW was significantly different while ligating the artery toward to the spleen in rats. Analysis of harmonics of BPW revealed that different organs might have their own natural frequency. It needs further studies to identify the relationship between individual organs and the harmonics so that we can interpret more from this present study.

There were some limitations to the study. Beside the inclusion and exclusion criteria, subjects without DM concern had low willingness to have blood tests that led to less amount of recruitment in control group. Furthermore, in addition to T2DM, some subjects also suffered from chronic diseases, such as chronic kidney disease, hypertension, hyperlipidemia, and others, because we enrolled the subjects from the outpatient department. We could not assess the effect of these chronic diseases in our current study. Further research can be done to clarify this question in the future. Even though we did not take these factors of chronic diseases into consideration, we were however still able to discriminate between T2DM and non-T2DM patients in the study.

## Conclusions

The study showed that the first and fifth harmonics (C_1_ and C_5_) differed significantly between the two patient groups, suggesting that analyzing the harmonic characteristics of non-invasively measured peripheral arterial pressure waveform may be a potential and easy-to-perform approach to discriminate T2DM-induced hemodynamic changes.

## Data Availability Statement

The datasets generated for this study are available on request to the corresponding author.

## Ethics Statement

The study involving human participants was reviewed and approved by the Research Ethics Committee, National Taiwan University Hospital (REC permit No. 1063704895) and the Institutional Review Board, Taipei Veterans General Hospital (IRB permit No. 1064903562). All participants provided their written informed consent before the study.

## Author Contributions

FL, J-ST, and S-CL contributed the conception and design of the study. J-ST, L-YL, and C-FL recruited the subjects. C-KL wrote the draft of the manuscript. C-KL, L-YL, C-FL, and T-WH organized the database and performed the statistical analysis. All authors contributed to the article and approved the submitted version.

## Conflict of Interest

The authors declare that the research was conducted in the absence of any commercial or financial relationships that could be construed as a potential conflict of interest.

## Footnotes

^1^NIH. *Who Is at Risk for Atherosclerosis?* Available online at: https://www.nhlbi.nih.gov/health-topics/atherosclerosis (accessed April 19, 2020).

## Abbreviations

T2DM: type 2 diabetes mellitus
BPW: blood pressure waveform
DM: diabetes mellitus.
